# Correction to: Long-term small-fiber neuropathy and pain sensitization in survivors of pediatric acute lymphoblastic leukemia after stem cell transplantation

**DOI:** 10.1007/s00432-021-03751-y

**Published:** 2021-08-13

**Authors:** Victoria Ruscher, Sascha Lieber, Jörn‑Sven Kühl, Johannes Schulte, Markus Blankenburg, Tobias Reindl, Pablo Hernáiz Driever

**Affiliations:** 1 Department of Pediatric Oncology/Hematology and Stem Cell Transplantation, Charité-Universitätsmedizin Berlin, Corporate Member of Freie Universität Berlin, Humboldt-Universität zu Berlin, and Berlin Institute of Health, Augustenburger Platz 1, 13353 Berlin, Germany; 2 Department of Pediatric Neurology, Psychosomatics and Pain Therapy, Klinikum Stuttgart–Olgahospital and Faculty of Health, Witten/Herdecke University, Witten, Germany

## Correction to: Journal of Cancer Research and Clinical Oncology (2020) 146:2143–2152 10.1007/s00432-020-03216-8

The article “Long-term small-fber neuropathy and pain sensitization in survivors of pediatric acute lymphoblastic leukemia after stem cell transplantation” written by Victoria Ruscher, Sascha Lieber, Jörn-Sven Kühl, Johannes Schulte, Markus Blankenburg, Tobias Reindl and Pablo Hernáiz Driever was originally published electronically on the publisher’s internet portal on April 28, 2020 without open access. With the author(s)’ decision to opt for Open Choice the copyright of the article changed to © The Author(s) 2020 and the article is forthwith distributed under a Creative Commons Attribution 4.0 International License, which permits use, sharing, adaptation, distribution and reproduction in any medium or format, as long as you give appropriate credit to the original author(s) and the source, provide a link to the Creative Commons licence, and indicate if changes were made. The images or other third-party material in this article are included in the article’s Creative Commons licence, unless indicated otherwise in a credit line to the material. If material is not included in the article’s Creative Commons licence and your intended use is not permitted by statutory regulation or exceeds the permitted use, you will need to obtain permission directly from the copyright holder. To view a copy of this licence, visit http://creativecommons.org/licenses/by/4.0/. Open Access funding enabled and organized by Projekt DEAL.

The original article has been updated.

